# The Pupillary Light Response Reveals the Focus of Covert Visual Attention

**DOI:** 10.1371/journal.pone.0078168

**Published:** 2013-10-29

**Authors:** Sebastiaan Mathôt, Lotje van der Linden, Jonathan Grainger, Françoise Vitu

**Affiliations:** Laboratoire de Psychologie Cognitive, Aix-Marseille Université, CNRS, Marseille, France; University of Muenster, Germany

## Abstract

The pupillary light response is often assumed to be a reflex that is not susceptible to cognitive influences. In line with recent converging evidence, we show that this reflexive view is incomplete, and that the pupillary light response is modulated by covert visual attention: Covertly attending to a bright area causes a pupillary constriction, relative to attending to a dark area under identical visual input. This attention-related modulation of the pupillary light response predicts cuing effects in behavior, and can be used as an index of how strongly participants attend to a particular location. Therefore, we suggest that pupil size may offer a new way to continuously track the focus of covert visual attention, without requiring a manual response from the participant. The theoretical implication of this finding is that the pupillary light response is neither fully reflexive, nor under complete voluntary control, but is instead best characterized as a stereotyped response to a voluntarily selected target. In this sense, the pupillary light response is similar to saccadic and smooth pursuit eye movements. Together, eye movements and the pupillary light response maximize visual acuity, stabilize visual input, and selectively filter visual information as it enters the eye.

## Introduction

The pupil adjusts its size in order to optimize visual acuity under varying levels of luminance [1], [2]. In darkness, acuity is limited by the number of photons that fall on the retina, and the pupil becomes enlarged to increase the influx of light. In brightness, acuity is limited by optical artifacts, and the pupil constricts to reduce spherical and chromatic aberrations. In addition, changes in pupil size reduce fluctuations in the amount of light that enters the eye, and thus constitute a first step in adaptation to darkness and brightness [3]. Stabilizing visual input and finding the optimal trade-off between sufficient light influx and minimal optical aberrations are among the primary functions of the pupillary light response (PLR).

The PLR is a constriction of the pupil in response to luminance increases. When going from brightness to darkness, the pupil gradually ‘unconstricts’ back to a resting state [4]. (For an animated example, see http://youtu.be/QLCxJKJMZuE.) Although this type of unconstriction leads to an enlargement of the pupil, it is very different from the dilation that occurs in response to a variety of cognitive and emotional stressors [5]. When a stressor is removed or when an organism habituates, the pupil gradually shrinks back to a resting state [6]. But this type of ‘undilation’ is fundamentally different from the constriction of the PLR.

In behavioral experiments, it is not possible to distinguish ‘undilation’ from constriction, or ‘unconstriction’ from dilation. However, lesion studies dating back to the 19^th^ century (reviewed in [7]) have shown a clear double dissociation. Lesions to the sympathetic nervous system selectively impair dilation in response to arousal. Reduced pupillary dilation is also observed in healthy elderly individuals and individuals suffering from Alzheimer’s disease, a finding that has been interpreted as reflecting an age- and disease-related impairment of the sympathetic nervous system [8], [9]. In contrast, lesions to the parasympathetic nervous system selectively impair the PLR. The distinction between pupillary dilation and the PLR has been confirmed in recent neurophysiological studies: Pupillary dilation is linked to activation of the Locus Coeruleus (LC), a brainstem area involved in regulating arousal and activity [10]. In contrast, the PLR may be linked to activation of the Superior Colliculus (SC) [cf. the effect of background luminance in 11], a midbrain area mostly known for its involvement in orienting responses and saccadic eye movements [12]. The distinction between dilation and constriction is evident even in the eye’s musculature: The PLR is driven primarily by the iris sphincter muscle, innervated by the oculomotor nerve, whereas dilation is driven primarily by the iris dilator muscle, innervated by the trigeminal nerve [13], [14].

The distinction between dilation and the PLR is important, because the two responses reflect very different processes [7]. Pupillary dilation has been variously described as reflecting mental effort [5], interest value [15], or “how intensely the system is processing” [16]. More generally, pupillary dilation occurs whenever an organism is somehow aroused, regardless of why and how [5], [17], [18]. Irene Loewenfeld aptly summarized this in her classic, but remarkably current review of the pupillary response: “Man may either blush or turn pale when emotionally agitated, but his pupils always dilate” [7].

Therefore, it is clear that pupil size, through dilation, reflects arousal and higher-level cognition. But the assumption has traditionally been that constriction occurs only in response to light, and is not affected by cognitive influences. (As is often the case with tacit assumptions, there are few direct references for this claim–only a remarkable paucity of claims to the contrary.) However, it has recently become clear that this assumption is incomplete.

A first indication that pupillary constriction may occur in response to things other than light comes from the finding that visual changes in structure, motion, or color trigger constriction as well. Strikingly, constriction occurs even when visual changes constitute an overall decrease in luminosity [19]–[22]. At first sight, this seems to conflict with the PLR, as one would expect the pupil to unconstrict in response to luminance decreases. But careful consideration shows that this apparent conflict is skin-deep. Visual changes are generally a mix of local increases and decreases in luminosity. Some parts of the image become brighter and induce a fast, active constriction, whereas other parts become darker and induce a slower, passive unconstriction. The combined effect is an initial constriction, which dissipates as unconstriction ‘catches up’. Therefore, the PLR can, in principle, account for pupillary constriction in response to most forms of visual change. However, it has been reported that a slight constriction is triggered even by visual changes that consist solely of local luminance decreases, such as the presentation of dark bars on a bright background [23]. This is not easily explained in terms of the PLR, although it is possible that in this case constriction is driven by local increases in perceived (but not objective) brightness [24]. Therefore, we believe that it is currently unclear how pupillary constriction in response to visual change relates to the PLR.

The second and more decisive indication that constriction is not purely a reflexive response to light comes from studies on binocular rivalry [25]–[30]. In binocular-rivalry experiments, both eyes are presented with conflicting stimuli, only one of which is consciously perceived at any one time. Already in 1966, Lowe and Ogle ( [25], for an even earlier report see [30)] reported a pupillary constriction when a participant’s percept switched from a dark stimulus in one eye to a bright stimulus in the other eye. This clearly showed that the PLR was not dependent solely on the amount of retinal illumination, but also on the participant’s visual awareness. This conclusion has recently gained additional support from studies showing that the PLR is sensitive to the perceived, rather than actual, brightness of a stimulus. The first to show this were Laeng and Endestad [24], who used brightness illusions to show that the pupil constricts more in response to stimuli that appear brighter, even when the actual luminance is controlled. In line with this result, Naber and Nakayama [31] and Binda and colleagues [32] subsequently showed that the pupil constricts in response to images of the sun, compared to equiluminant images without a sun. Taken together, these studies do not call into question that constriction results from luminance increases, but they do show that the strength of constriction can be modulated by higher-level cognition, or by the interpretation that is attached to visual input. Thus, unlike traditionally assumed, the PLR is not merely a reflexive response to retinal illumination.

Finally, in a related study, Binda and colleagues [33] showed that the PLR is modulated by covert visual attention. That is, the PLR is driven primarily by objects that we attend to, even if we don’t look directly at these objects. In Binda et al.’s [33] study, participants covertly attended to one of two large disks, which were presented on the left and right sides of the screen. Participants kept their eyes fixated on a central point, and never looked directly at either disk. Both disks contained a small dot, and the participant’s task was to count the number of times that the dot in the attended disk changed color. The crucial manipulation was that the attended disk was either bright or dark. The researchers’ prediction was straight-forward and confirmed by the results: Attending to the bright disk should, and did, trigger a pupillary constriction relative to attending to the dark disk. This finding fits very well with the presumed function of the PLR as a way to optimize visual acuity, because optimal pupil size is different for the different objects that are within our field of view at any one time. For example, when typing, you may keep your eyes on the screen in front of you, while shifting your attention back and forth between the screen and the keyboard underneath. In this case, optimal perception of the keyboard, which is relatively dark, requires a larger pupil than optimal perception of the screen, which is bright. The study by Binda and colleagues [33] and the findings that we report here suggest that, in principle, it is possible to determine whether you are attending to the screen or the keyboard by monitoring the size of your pupil.

Here we follow up on recent studies that have shown a modulation of the PLR by cognitive factors [24], [31], [32] and in particular by covert visual attention [33]. We used a paradigm that was similar to the one used by Binda and colleagues [33], but allowed us to link the pupillary response more directly to behavior. In addition, in Exp. 2 we equated the difficulty between the attend-to-bright and attend-to-dark conditions, to avoid a potentially confounding effect of task difficulty on pupil size [5]. We conducted two experiments, which were variations on the classic Posner-cueing (or attentional-cueing) paradigm [34]. Participants viewed stimuli on a computer screen, while their gaze remained fixated on a central dot. The screen was divided into a bright and a dark half (bright left, dark right, or vice versa), separated by a central gray band that contained the fixation dot. Participants reported the orientation of a peripheral target stimulus that was presented to the left or right, on the dark or bright background. Before the target appeared, a cue indicated its probable location (Exp. 1: a leftwards−/rightwards-pointing arrow, 75% valid; Exp. 2: a left−/right-panned voice saying “*left*” or “*right*”, 80% valid).

## Results

### Statistical Analysis

Except stated otherwise, we used linear mixed-effects modeling (LMM) with participant as random effect. Markov chain Monte Carlo simulation (MCMC) was used to estimate *p*-values and 95% confidence intervals (CIs) [35]. CIs are derived from fixed-effect slope CIs, such that non-overlapping error bars indicate *p*<.05.

### Behavioral Cueing Effect

**Figure 1:**
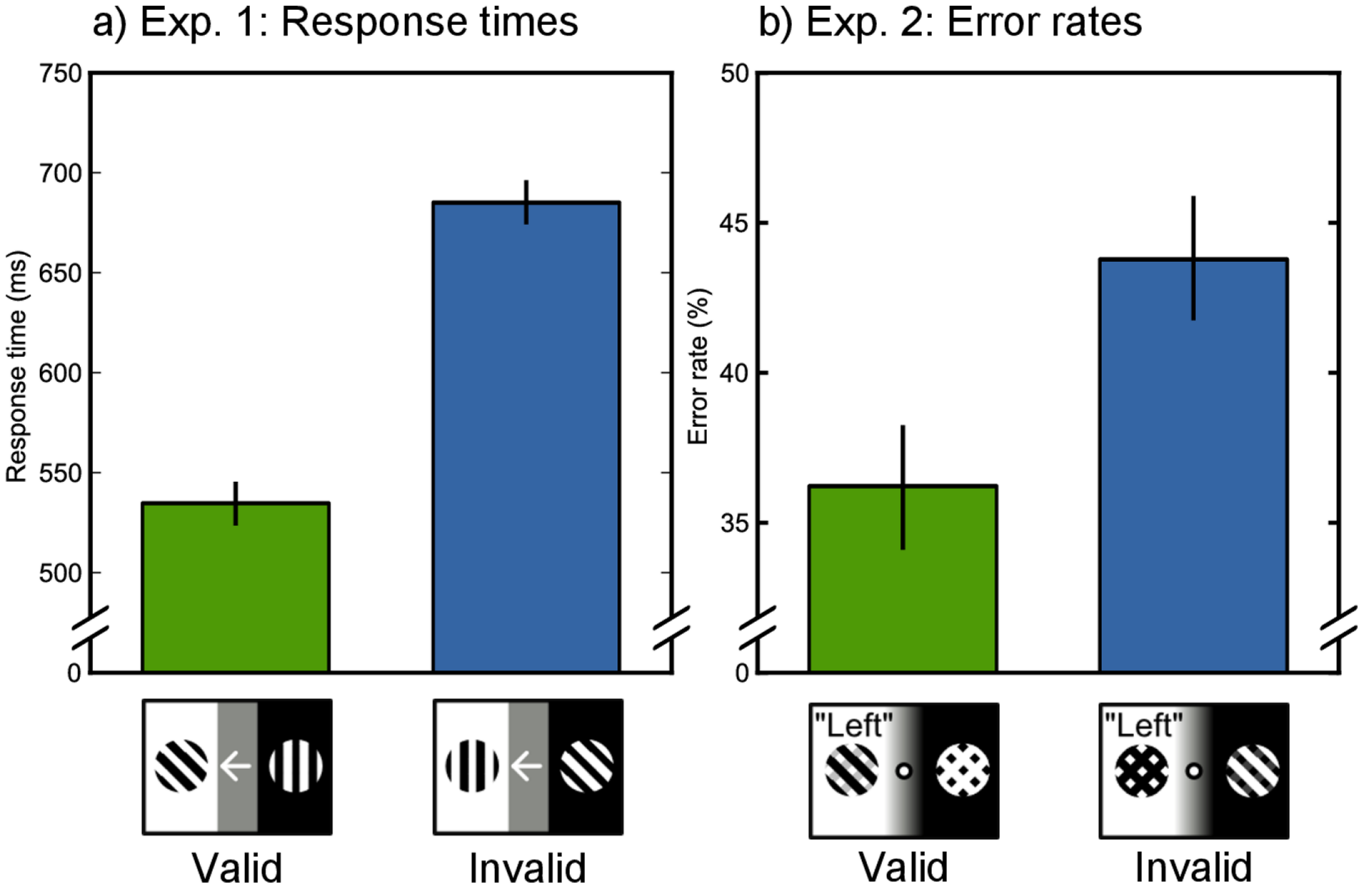
Behavioral results. a) In Exp. 1, responses were faster on validly cued trials, compared to invalidly cued trials. b) In Exp. 2, error rates were lower on validly cued trials, compared to invalidly cued trials.

Both Exp. 1 and Exp. 2 revealed an effect of cue validity (Fig. 1); on response times (Exp. 1, valid: *N* = 1047, *M* = 535 ms, invalid: *N* = 320, *M* = 685 ms, *t* = 14.7, *p*<.0001) and error rate (Exp. 1, valid: *M* = 11%, invalid: *M* = 20%, *t* = 3.6, *p* = .0003; Exp. 2, valid: *N* = 2576, *M* = 36%, invalid: *N* = 651, *M* = 44%, *t* = 3.6, *p* = .0004).

### Modulation of the PLR by Covert Visual Attention

**Figure 2:**
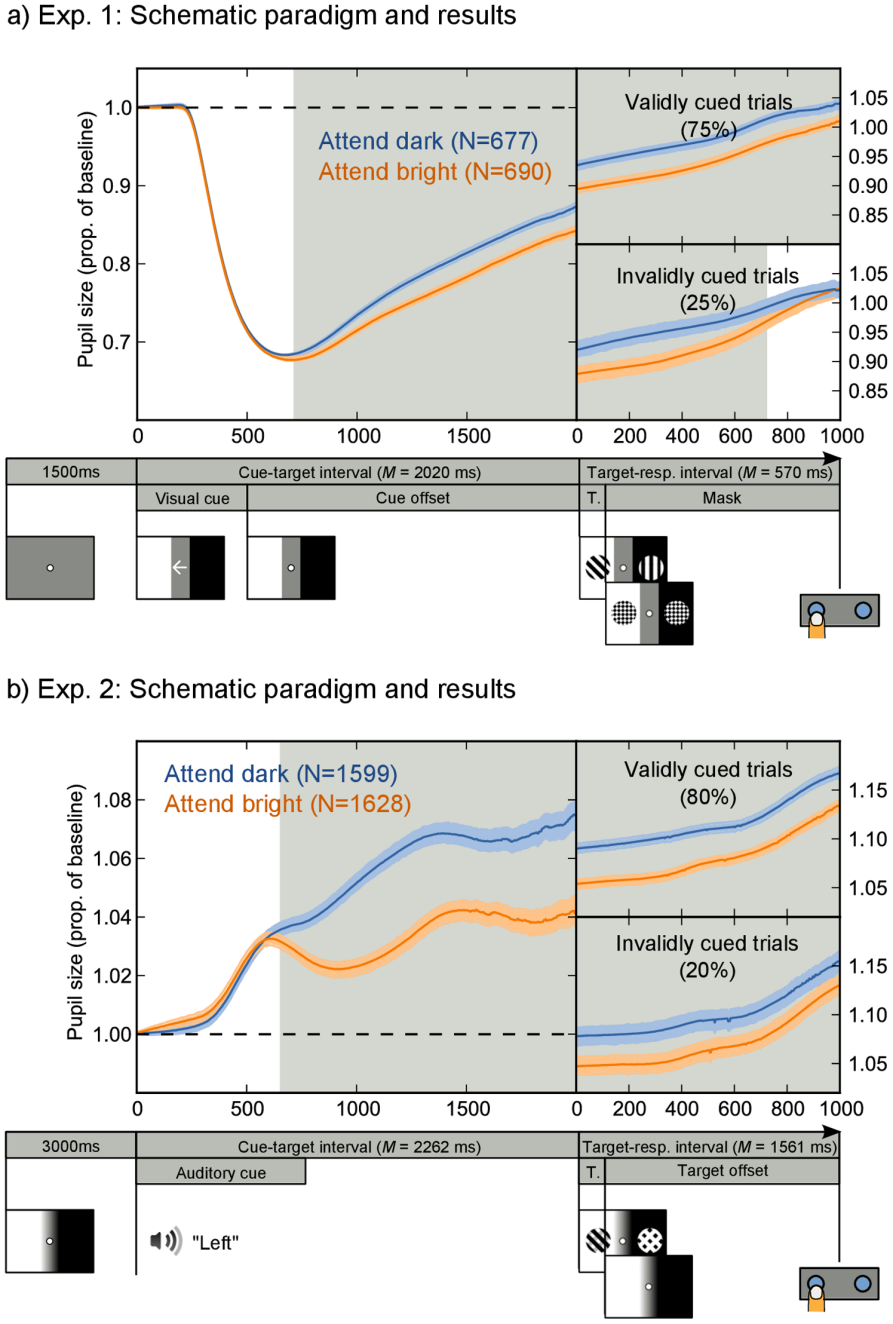
Schematic paradigm and pupil-size traces for Exp. 1 (a) and Exp. 2 (b). Pupil size was larger on attend-dark than on attend-bright trials, which reflects attentional modulation of the PLR. This modulation was evident from 717 ms (in Exp 1) and 656 ms (in Exp. 2) after the presentation of the cue, as indicated by the gray shading. In Exp. 1 there was also an overall constriction in response to the (visual) cue, with a mean latency of 241 ms.

The main results are shown in Fig. 2, in which mean pupil size is plotted over time. As predicted, the pupil was smaller in attend-bright trials than in attend-dark trials. In Exp. 1, the cue was a visual change and the overall response was a combination of fast visual-change-induced constriction [19], with a mean response latency of 241 ms (*SD* = 25; based on a.5 pupil-size/s velocity threshold) [4], and slower dilation. The dilation observed here was presumably a mix of task-evoked dilation [5] and recovery (‘unconstriction’) from the visual-change-related constriction. In Exp. 2, the cue was auditory, and the overall response was only task-evoked dilation. Crucially, in both experiments the pupillary response diverged between the attend-bright and attend-black conditions from respectively 716 ms (Exp. 1) and 656 ms (Exp. 2) post cue. These latencies presumably reflect the inherent latency of the PLR (approx. 200–500 ms [4]), and the time required for processing the cue and orienting attention. Strikingly, in Exp. 2 the pupil went into a period of constriction in the attend-bright condition, while continuing to dilate in the attend-dark condition, showing that attentional modulation can qualitatively alter the pupillary response. In both experiments, pupil-size modulation was long-lasting and corresponded to a relative pupil-area difference of 3–4%. This effect appears to be slightly smaller in size than that reported by Binda and colleagues [33]. If we assume a resting-state pupil diameter of 5 mm (not reported, cf. [36]), they observed a pupil-area difference of about 8%. The difference in effect size between their study and ours may be due to a variety of factors, such as task differences or the fact that the bright stimuli used by Binda and colleagues were more luminous than ours (105 cd/m^2^ vs 96 cd/m^2^).

In Exp. 1, pupil-size modulation tended to dissipate after the appearance of the target on invalidly cued trials, suggesting that participants shifted their attention away from the cued side, towards the side where the target had appeared. However, we note this point here only for completeness, because this tendency was weak and not statistically reliable.

#### Relationship between pupil-size modulation and behavioral cueing effect

We reasoned that modulation of pupil size by attention should be stronger on trials where participants attended more strongly to the cued side. Because the primary behavioral measure differed between the two experiments, we used a slightly different approach to test this prediction in each experiment.

**Figure 3:**
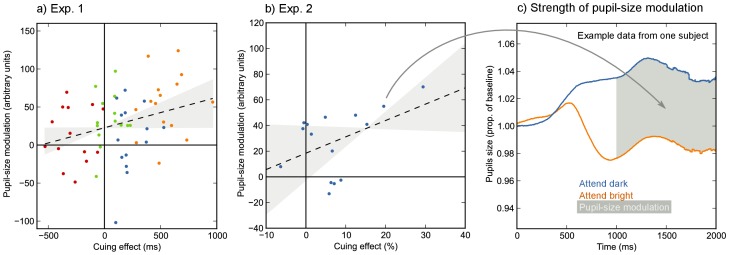
The strength of the pupil-size modulation is related to the strength of the behavioral cueing effect in response times (a; Exp. 1) and accuracy (b; Exp. 2). a) Dots indicate participant bin means. Colors indicate different bins (see text for details). Regression line and slope CI are estimated with LMM and MCMC. b) Dots indicate participant means. Regression line and slope CI are estimated with linear regression. c) The strength of the pupil-size modulation as shown in a) and b) corresponds to the surface area between the attend-dark and attend-bright pupil-size traces in the 1000–2000 ms interval of the cue-target epoch. Intervals where the attend-bright trace fell above the attend-dark trace were counted as negative surface. The example shown here corresponds to a single data point in b), as indicated.

In Exp. 1, where the primary behavioral measure was RT, attending strongly to the cued side should lead to faster responses on validly cued trials, and slower responses on invalidly cued trials. The cuing effect is therefore defined as M(RT)_invalid_ - M(RT)_valid_. For each participant separately, we rank ordered all validly cued trials based on RT from fast to slow and divided the resulting list into four bins. All invalidly cued trials were rank ordered from slow to fast and similarly divided into four bins. The fastest validly cued bin was paired with the slowest invalidly cued bin, thus leading to a very large cueing effect in that subset of trials (the orange dots in Fig. 3a). Conversely, the slowest validly cued bin was paired with the fastest invalidly cued bin, thus leading to a negligible or even inverse cueing effect in that subset of trials (the red dots in Fig. 3a). Next, we determined the strength of the pupil-size modulation for each bin. The strength of the pupil-size modulation was determined by taking the area between the average pupil-size traces for the attend-dark and attend-bright trials in the 1000–2000 ms window of the cue-target epoch, such that a positive area corresponds to a modulation in the predicted direction (for an example, see Fig. 3c). A LMM with pupil-size modulation as dependent measure, cueing effect as fixed effect, and participant as random effect, revealed a relationship between cueing effect and pupil-size modulation, *t* = 2.7, *p* = .0101 (Fig. 3a). This indicates that, as predicted, stronger modulation of the PLR is accompanied by a stronger behavioral cueing effect.

In Exp. 2, where the primary behavioral measure was accuracy, attending strongly to the cued side should lead to higher accuracy on validly cued trials, and lower accuracy on invalidly cued trials. Since accuracy is, for individual trials, a dichotomous measure (i.e. a response is correct or not), we could not employ the rank-ordering procedure used in Exp. 1. Instead, we simply calculated the cuing effect (100*M(correct)_valid_ - 100*M(correct)_invalid_) and the strength of the pupil-size modulation for each participant. Because there were no random effects, we determined a between-subjects correlation. We found a positive correlation, albeit less reliable than the LMM results of Exp. 1, between the cuing effect and the strength of the pupil-size modulation, *r* = .45, *p* = .0931 (Fig. 3b).

### Effect Size: Pupil-size Modulation versus Behavioral Measures

For both experiments, we determined the effect size (Cohen’s *d*) for the primary behavioral measure and the attentional modulation of the PLR. For Exp. 1, we determined, per participant, the behavioral cuing effect in response times (M(RT)_invalid_ - M(RT)_valid_) and the strength of the pupil-size modulation (as in Fig. 3c). We determined Cohen’s *d* for the effect size compared to 0, and found *d* = 1.14 for the behavioral cuing effect, and *d* = 1.07 for the pupil-size modulation. For Exp. 2, we conducted a similar analysis using accuracy for the behavioral cuing effect (100*M(correct)_valid_ - 100*M(correct)_invalid_), and found *d* = 0.84 for the behavioral cuing effect, and *d* = 1.12 for the pupil-size modulation. Clearly, the effect size of attentional pupil-size modulation is large (>0.8) and comparable to the effect size of behavioral measures that are typically used as indices of attention.

## Discussion

We have shown that attending to a bright area induces a pupillary constriction relative to attending to a dark area under identical visual input (Fig. 2). Phrased differently, we found that the pupillary light response (PLR) was modulated by covert visual attention, consistent with a recent report by Binda and colleagues [33]. This modulation arose about 716 ms (Exp. 1) and 656 ms (Exp. 2) after the presentation of the cue and was a strong effect (Cohen’s *d* >1). In one experiment, the pupillary constriction that resulted from attending to a bright area even temporarily reversed the overall task-evoked dilation (Fig. 2b). Furthermore, we have shown for the first time that attentional modulation of the PLR predicts behavioral cueing effects in response times (Fig. 3a) and, although less reliably, in accuracy (Fig. 3b). These findings, and recent studies that show modulation of the PLR by recognition of the sun [31], [32], perceived brightness [24], and binocular rivalry ([26], [31] for related findings see [25], [28]–[30]) have overturned the long-standing notion that the pupillary response to light is purely a reflex in response to retinal illumination.

### Pupil Size as a Novel way to Track the Focus of Attention

Our findings suggest that pupil size can be used to track the focus of attention continuously and on-line, without the need for collecting manual responses from the participant. This opens up a wide range of experimental possibilities. For example, Treisman and Gelade [37] famously proposed a distinction between serial and parallel visual search (i.e. finding a target among multiple distractors). In serial search, stimuli are covertly (assuming that eye movements are not allowed) scanned one at a time until the target is found. In parallel search, the target ‘pops out’ and no serial scanning is required. Support for this distinction comes largely from experiments that have measured behavioral response times. Thus, the existence of a covert serial scanning mechanism has been inferred from indirect evidence, most of which is also consistent with models that do not rely on purely serial processing (e.g., [38]). In light of the present results, it may be possible to track the participant’s focus of attention as he or she covertly scans a search display, and obtain direct evidence for (or against) a serial scanning mechanism in the absence of eye movements (see also [39]). This approach complements a pupil-size-deconvolution method that has recently been described and can be used to track the susceptibility to sensory input over time [40], [41]. Phrased differently, pupil size does not only reveal *whether* one is paying attention (temporal attention [40], [41]), but also *what* one is paying attention to (visuospatial attention [33]; the present study). The extent to which this approach will be feasible in practice remains to be determined, and experiments will likely be constrained to relatively simple displays with large differences in luminosity. Nevertheless, we are excited about the possibilities that these new pupillometry-based methods offer.

### Early Selection by Covert Visual Attention

The effects of attention on visual perception are pervasive (for reviews, see [42], [43]). Attention increases the perceived brightness of stimuli [44], and people respond more quickly and accurately to stimuli that are in the focus of attention [34]. In fact, objects that are not within the focus of attention are frequently not detected at all, a phenomenon called inattentional blindness [45]. From a neural perspective, attended stimuli elicit a more vigorous and selective response throughout the visual cortex [46], [47]. With respect to the present study, the finding that visual attention modulates the PLR extends the reach of visual attention even further, to the input stage of visual perception [33]. By adjusting the size of the pupil to the brightness of objects that are in the focus of attention, the selection process that characterizes visual perception begins as soon as light enters the eye. The PLR is thus a mechanism for early selection, and constitutes a first step in the visual-processing hierarchy [48].

### The Pupillary Light Response in Relation to Spatial Eye Movements

Changes in pupil size are rarely considered in discussions of spatial eye movements, such as saccades and smooth pursuit (but see [49], [50]). This disconnect is evident even in the existence of two largely separated fields of research: Pupillometry is concerned with changes in pupil size, whereas eye-movement research is primarily concerned with saccades and smooth pursuit. Anecdotally, the word ‘pupil’ occurs only once in a classic review of eye-movement research (in the context of the methodology required to measure spatial eye movements [51]) and not at all in a recent leading review of the field [52].

Yet we believe that the present results, as well as the studies described in the introduction [24]–[33], are best understood by considering the PLR as a type of eye movement that is closely related to spatial eye movements. Firstly, both spatial eye movements and the PLR optimize visual acuity. Spatial eye movements do so by bringing the object of interest into view of the high-resolution foveal part of the retina. The PLR does so by balancing sufficient light influx against spherical and optical aberrations [1], [2]. Secondly, both spatial eye movements and the PLR play a role in stabilizing visual input. Spatial eye movements stabilize visual input during external or self-generated movement, for example by keeping your eyes fixated on an object while you shake your head [42], [53]–[55]. The PLR similarly stabilizes visual input, by keeping the amount of light that enters the eye relatively stable during luminance changes of the environment [3]. Thirdly, microstimulation of the superior colliculus (SC), a midbrain structure, elicits both saccadic eye movements [12] and pupillary dilation ([11], see also [56]), showing that these movements are mediated by overlapping neural pathways. (We are not aware of studies showing directly that SC-microstimulation elicits smooth pursuit, but there is substantial evidence that the SC is involved in smooth pursuit as well [57].).

Finally, neither spatial eye movements nor the PLR are under full voluntary control, yet they can all be indirectly controlled through covert visual attention. This last point is perhaps best illustrated by analogy with smooth pursuit. A smooth pursuit eye movement is essentially an encephalized form of the optokinetic reflex (OKR), a gaze-stabilizing reflex that keeps your eyes ‘glued’ to the visual field [58]. The OKR is a stereotyped movement over which we have little control: If you look out the window of a moving train, you cannot help but track the passing landscape with your eyes. The only way in which you can control the OKR is by focusing on a specific object. To stick with our example, you could attend to a scratch on the window, in which case your eyes would cease to track the landscape behind it and stay fixated on the scratch. Similarly, you cannot make smooth pursuit eye movements across a clear and unmoving sky–unless there is a bird to track with your eyes. In other words, smooth pursuit is essentially a reflex that is modulated by attention: You can control the target of the movement, but you cannot control the movement itself. The situation appears to be analogous for the PLR. The PLR is a reflex in the sense that the basic movement is beyond voluntary control. No matter how much willpower you exert, the PLR will always be a constriction in response to a bright stimulus. However, by focusing your attention you can control which object is ‘tracked’ by the PLR. More generally, the PLR, saccades, and smooth pursuit are all best characterized as stereotyped responses to targets that can be voluntarily selected. An important direction for future research will be to systematically investigate the link between the PLR and spatial eye movements, and to investigate to which extent these rely on common cognitive and neural mechanisms.

## Conclusion

In conclusion, we have shown that the pupillary light response (PLR) is modulated by the brightness of covertly attended objects, while visual input is kept constant. This finding is consistent with the pervasive effects of visual attention on early neural activity [46], [47], [59] and perception [43], [44], and recent reports that the PLR is modulated by higher-level cognition [24], [31]–[33]. By measuring both manual responses and pupil-size modulation in a classic Posner-cuing paradigm, we have shown that both behavioral and pupil-size measures can be used effectively to track the focus of attention. However, unlike manual responses, pupil size is a continuous measure that does not interfere with the participant’s task. Therefore, we believe that pupil-size measures may offer a viable new tool for visual-attention research. Finally, we have argued that pupillary responses and spatial eye movements, such as saccades and smooth pursuit, are interconnected forms of movement, despite being the focus of largely separated fields of research. Together, eye movements and pupillary responses allow us to optimize visual acuity, stabilize visual input, and focus on specific objects.

## Methods

### Materials

Participant data and experimental scripts are available from the first author’s website or from <https://github.com/smathot/data_repository>.

### Participants and Ethics Statement

Fifteen observers (thirteen naive participants and two authors; ten women) participated in Exp. 1. Fifteen observers (thirteen naive participants, five of whom had participated in Exp. 1, and two authors; ten women) participated in Exp. 2. Participants were recruited through the subject pool of Aix-Marseille Université. All participants were between 18 and 40 years of age and reported normal or corrected visual acuity. All participants provided digital informed consent through a computerized consent form that was presented prior to the experiment. Participants who were students of the university received monetary compensation or course credit, and signed an additional written consent form. The experiment was conducted with approval of the local ethics committee of Aix-Marseille Université, and was in accordance with the declaration of Helsinki.

### Apparatus

The right eye was recorded with an EyeLink 1000 (SR Research, Mississauga, Canada, ON), a video-based eye tracker sampling at 1000 Hz. Stimuli were presented on a 21″ CRT monitor (1024×768 px, 100 Hz). Stimulus presentation was controlled with OpenSesame [60]/PsychoPy [61].

### Experiment 1: Procedure and Stimuli

**Figure 4:**
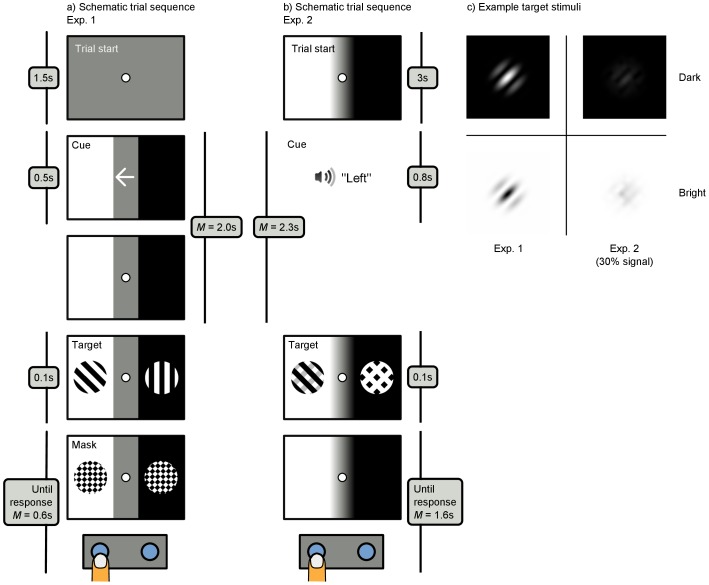
a, b) Schematic example trials for Exp. 1 and Exp. 2 (see text for details). c) Example target stimuli. The mean signal-to-noise ratios in Exp. 2 were 19.8% (on dark background) and 17.8% (on bright background).

Before each trial, a 1-point eye-tracker recalibration was performed (“drift correction”). Each trial started with the presentation of a central bright (96.0 cd/m^2^) fixation dot on a gray background (21.9 cd/m^2^) for 1500 ms (Fig. 4a). Next, a visual cue (a central white arrow pointing left or right) was presented for 500 ms, indicating the probable location of the target (75% validity). Simultaneously with the cue, the display was divided into a bright (96.0 cd/m^2^) and a dark (0.7 cd/m^2^) half, separated by a central gray band (21.9 cd/m^2^, 7.3° wide). Following a random interval (*µ* = 2500 ms, *σ* = 500 ms, from a normal distribution, min. = 1500 ms) after the onset of the cue, the target was presented 9.4° to the left or the right of the fixation dot. The target was a Gabor patch (*sf* = 1.7 cycles/°, σ = 0.71°, contrast = 100%) rotated 45° clockwise/counterclockwise from a vertical orientation (Fig. 4c). A distractor (a vertically oriented Gabor patch) was presented at the location opposite from the target. After 100 ms, the target and distractor were masked by patches of white noise with a Gaussian envelope (σ = 0.71°). The mask was shown until the participant reported the orientation of the target by pressing one of two buttons with their left (counterclockwise target) or right (clockwise target) hand. Participants were instructed to respond as fast as possible while maintaining 80–90% accuracy. All experimental factors (cue side, cue validity, left/right brightness) were randomized within blocks. The experiment consisted of 160 trials (10 blocks), preceded by 16 practice trials.

### Experiment 2: Procedure and Stimuli

The primary aim of Exp. 2 was to replicate the pupil-size modulation observed in Exp. 1 in a different paradigm. Furthermore, we controlled on-line for fixational eye movements (thus allowing us to retain more usable data than in Exp. 1), used an auditory cue to avoid the cue-related constriction observed in Exp. 1, and equated the difficulty of targets presented on dark and bright backgrounds.

The paradigm of Exp. 2 was similar to that of Exp. 1, with the following exceptions (Fig. 4b). Throughout each trial, the display was divided into a bright and a dark half, separated by a central gray band (5.6° wide). A continuous gaze-contingent algorithm maintained the gray band centered on the participant’s point of gaze, so that fixational eye movements did not bring the eyes closer to the bright or dark area. The maximum displacement of the gray band was 2.8° (i.e. no compensation occurred for larger eye movements). To reduce the visibility of the visual jitter that resulted from this procedure, a luminance gradient separated the gray band and the flanking bright and dark areas. Each trial started with a central fixation dot. After 3000 ms, an auditory cue indicated the probable target location (80% validity). To maximize the cueing effect, the cue was both endogenous (i.e. the word ‘*left*’/‘*right*’ or ‘*gauche*’/‘*droite*’, depending on the preferred language of the participant) and exogenous (i.e. panned left or right in the headphones), and was 100% valid during the practice phase. After a random interval (sampled from a 1500–3000 ms uniform distribution) the target was presented. The target was a mixture between signal (a left−/rightwards tilted Gabor patch, *sf* = 2.1 cycles/°, σ = 0.5°, contrast = 20%) and noise with the same spatial frequency as the signal (Fig. 4c). At the location opposite from the target, a pure-noise distractor was presented. After 100 ms, the target and distractor were removed (not masked). Participants were instructed to respond as accurately as possible at their preferred pace. Accuracy on validly cued trials was maintained at approximately 66%, separately for targets presented on bright and dark backgrounds, by adjusting the signal-to-noise ratio of the target with a 2-up-1-down staircase procedure with 5% steps. The experiment consisted of 240 trials (6 blocks), preceded by 40 practice trials.

### Pupil-trace Analysis

**Figure 5:**
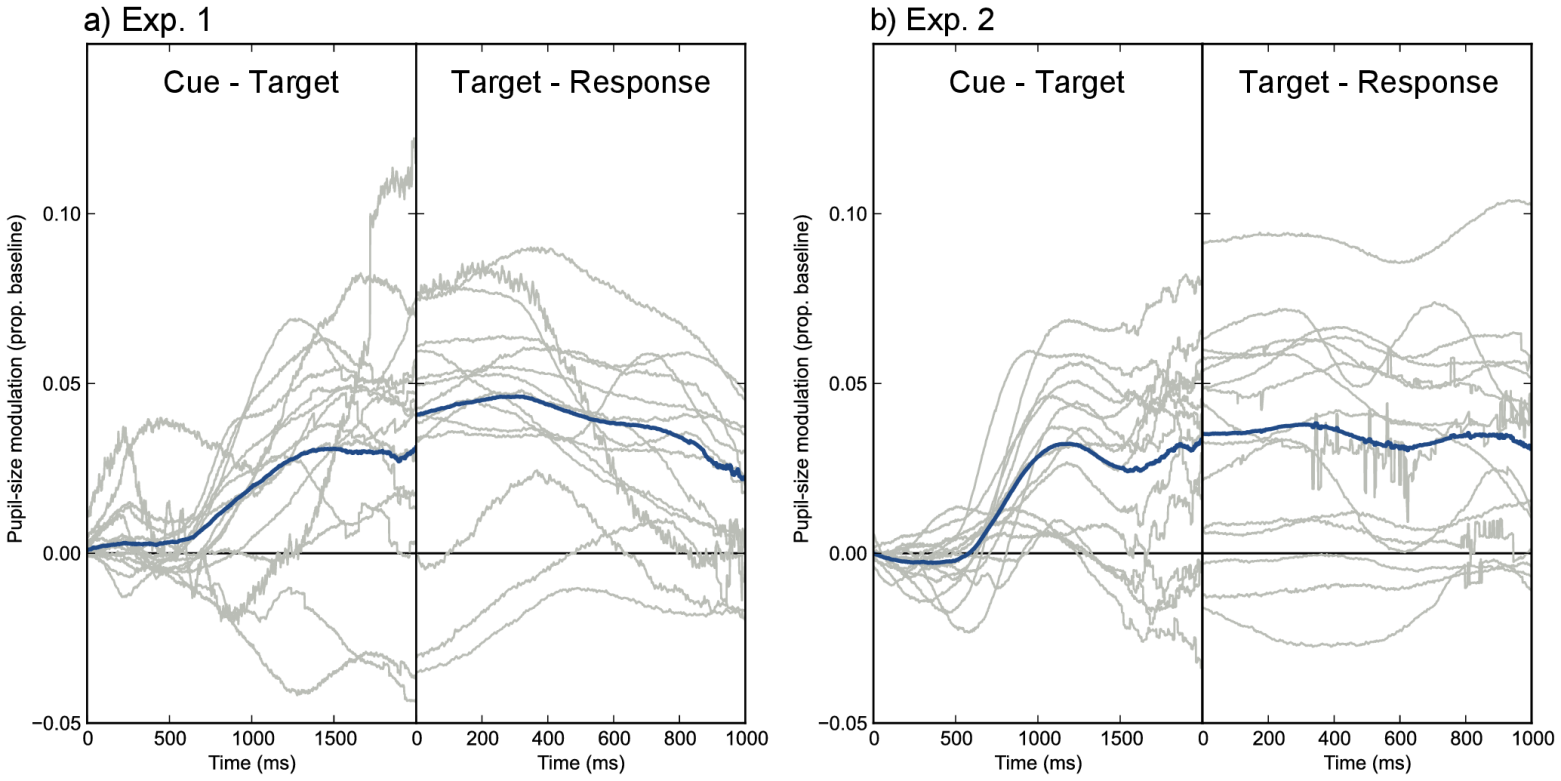
Pupil-size modulation (pupil size in attend-dark trials minus pupil size in attend-bright trials) during the cue-target and target-response epochs from Exp. 1 (a) and Exp. 2 (b). Gray lines correspond to individual-participant traces. Blue lines are grand-mean traces.

Each trial was divided into three epochs: the baseline epoch, the cue-target epoch, and the target-response epoch. The baseline epoch spanned the 100 ms prior to the presentation of the cue. Because the cue-target interval was jittered, and because response times are inherently variable, the cue-target and target-response epochs varied in length. Therefore, in Fig. 2 and Fig. 5, the right-most parts of the average pupil traces are based on fewer data points than the left-most parts. Pupil size was based on pupil surface and is reported relative to the mean pupil size during the baseline epoch. No signal smoothing was applied, except for the purpose of blink removal (see below). For each time point, we determined the effect of condition (attend-bright vs attend-dark) on pupil size, using a significance threshold of *p*<.05 for 200 consecutive samples. The first significant sample was used as the onset of divergence between attend-bright and attend-dark trials.

### Blinks, Gaze Errors, Fixational Eye Movements, and Outlier Removal

Pupil size during blinks was reconstructed using cubic-spline interpolation [62]. Blink frequency did not vary systematically between attend-bright and attend-dark trials (Exp. 1: *t* = 1.64, *p* = .1005; Exp. 2: *t* = 0.23, *p* = .8166). In Exp. 1, trials were discarded if at any point during the trial a single gaze sample deviated more than 2° from the fixation dot (33.2%). Because Exp. 1 was a speeded RT task, trials were discarded when the RT was more than 2.5 *SD*s above or below the participant’s mean RT (2.0%). Finally, trials were discarded when pupil size (averaged across the trial) deviated more than 2.5 *SD*s from the participant’s mean pupil size (1.1%). In Exp. 2, trials were discarded when horizontal gaze deviation exceeded the maximum displacement of the central gray bar (1.9%). Because Exp. 2 was a non-speeded task, trials were discarded only when the participant did not respond within 5000 ms (0.1%), and when pupil-size deviated more than 2.5 *SD* from the participant’s mean (2.4%).

**Figure 6:**
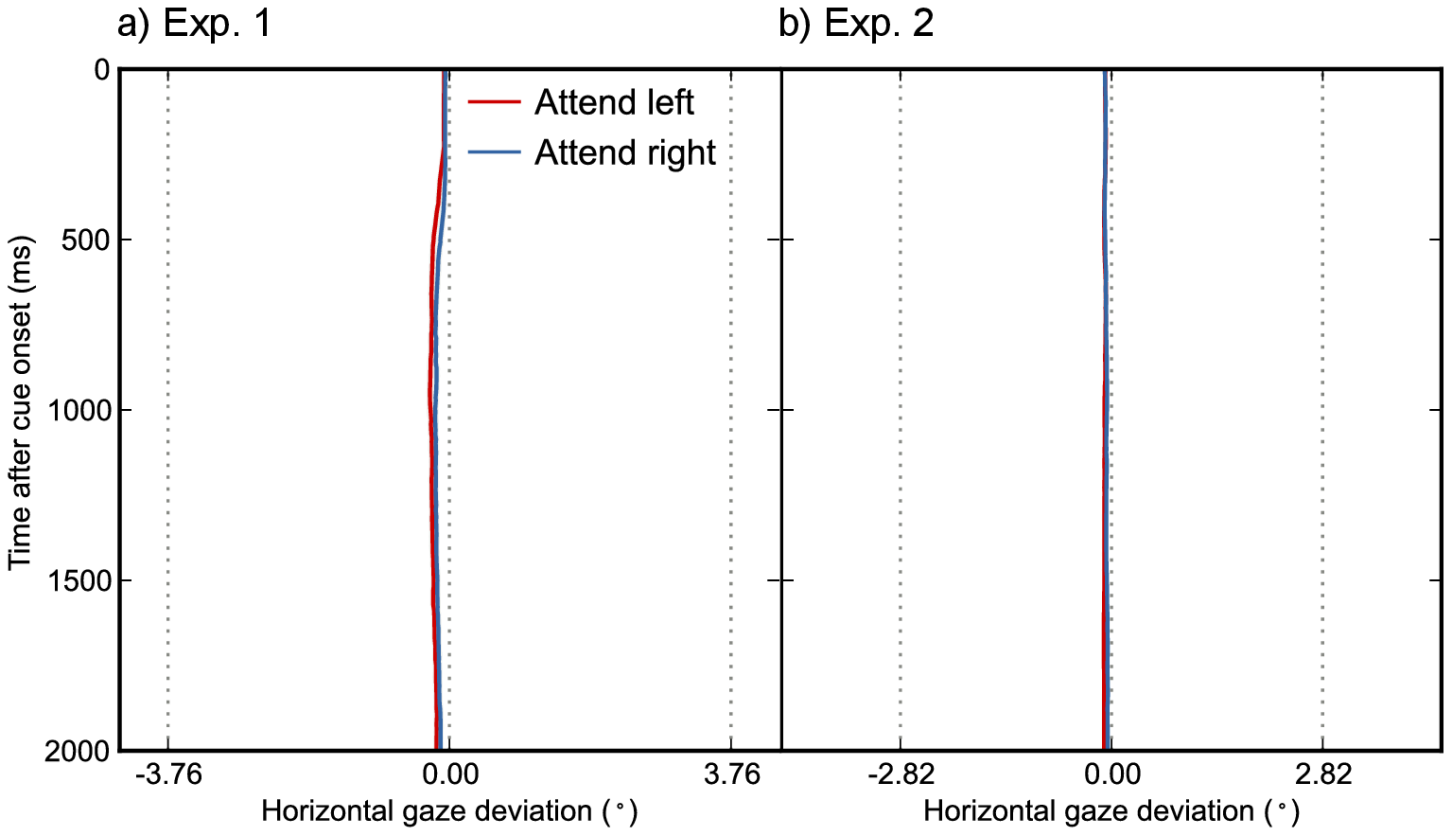
Horizontal gaze position over time for Exp. 1 (a) and Exp. 2 (b). Colored lines indicate the mean horizontal gaze deviation across participants. Central dotted lines indicate the display center. Peripheral dotted lines, respectively 3.76° and 2.82° from the display center, indicate the edges of the central gray shading (see Fig. 4). There was a slight tendency for the eyes to fixate leftwards of the display center, but no discernible tendency for gaze to gravitate towards the focus of attention. (In addition, for Exp. 2, any systematic gaze deviation would have been compensated for by the gaze-contingent algorithm, described in the main text.).

Even when no saccadic eye movements are made, fixational eye movements gravitate towards the focus of attention [63]. In Exp. 1, we controlled for this by calculating the average horizontal gaze bias during the cue-target epoch for each trial. Every trial was paired with another trial that had an opposite but approximately equal bias (a difference of less than 0.14°). All trials that could not be paired were discarded (6.6%), thus eliminating directional bias in fixational eye movements. In Exp. 2, we controlled for fixational eye movements through an on-line gaze-contingent algorithm that locked the horizontal position of the gray bar to the point of gaze as described above. For trials included in the analysis, there was no discernible tendency for the eyes to gravitate towards the attended side (see Fig. 6).

After selection, there were 1367 (Exp. 1) and 3227 (Exp. 2) pupil-size traces for further analysis.

## References

[1] Woodhouse JM (1975) The effect of pupil size on grating detection at various contrast levels. Vision Res 15: 645–648 10.1016/0042-6989(75)90278-3 1138478

[2] Campbell FW, Gregory AH (1960) Effect of size of pupil on visual acuity. Nature 4743: 1121–1123 10.1038/1871121c0 13690234

[3] Woodhouse JM, Campbell FW (1975) The role of the pupil light reflex in aiding adaptation to the dark. Vision Res 15: 649–653 10.1016/0042-6989(75)90279-5 1138479

[4] Ellis CJ (1981) The pupillary light reflex in normal subjects. Br J Ophthalmol 65: 754–759 10.1136/bjo.65.11.754 7326222 PMC1039657

[5] Beatty J (1982) Task-evoked pupillary responses, processing load, and the structure of processing resources. Psychol Bull 91: 276–292 10.1037/0033-2909.91.2.276 7071262

[6] Lowenstein O, Loewenfeld IE (1950) Role of sympathetic and parasympathetic systems in reflex dilatation of the pupil: Pupillographic studies. Arch Neurol Psychiatry 64: 313–340 10.1001/archneurpsyc.1950.02310270002001 15433651

[7] Loewenfeld IE (1958) Mechanisms of reflex dilatation of the pupil. Doc Ophthalmol 12: 185–448 10.1007/BF00913471 13609524

[8] Prettyman R, Bitsios P, Szabadi E (1997) Altered pupillary size and darkness and light reflexes in Alzheimer’s disease. J Neurol Neurosurg Psychiatry 62: 665–668 10.1136/jnnp.62.6.665 9219763 PMC1074161

[9] Bitsios P, Prettyman R, Szabadi E (1996) Changes in autonomic function with age: a study of pupillary kinetics in healthy young and old people. Age Ageing 25: 432–438 10.1093/ageing/25.6.432 9003878

[10] Aston-Jones G, Cohen JD (2005) An integrative theory of locus coeruleus-norepinephrine function: adaptive gain and optimal performance. Annu Rev Neurosci 28: 403–450 10.1146/annurev.neuro.28.061604.135709 16022602

[11] Wang CA, Boehnke SE, White BJ, Munoz DP (2012) Microstimulation of the monkey superior colliculus induces pupil dilation without evoking saccades. J Neurosci 32: 3629–3636 10.1523/JNEUROSCI.5512-11.2012 22423086 PMC6703448

[12] Robinson DA (1972) Eye movements evoked by collicular stimulation in the alert monkey. Vision Res 12: 1795–1808 10.1016/0042-6989(72)90070-3 4627952

[13] Betts JG, DeSaix P, Johnson E, Johnson JE, Korol O, et al (2013) Anatomy and Physiology. Houston, TX: OpenStax College. Available: http://openstaxcollege.org/textbooks/anatomy-and-physiology. Accessed 2013 Aug 1.

[14] Tanaka T, Kuchiiwa S, Izumi H (2005) Parasympathetic mediated pupillary dilation elicited by lingual nerve stimulation in cats. Invest Ophthalmol Vis Sci 46: 4267–4274 10.1167/iovs.05-0088 16249507

[15] Hess EH, Polt JM (1960) Pupil size as related to interest value of visual stimuli. Science 132: 349–350 10.1126/science.132.3423.349 14401489

[16] Just MA, Carpenter PA (1993) The intensity dimension of thought: Pupillometric indices of sentence processing. Can J Exp Psychol 47: 310–339 10.1037/h0078820 8364533

[17] Laeng B, Sirois S, Gredebäck G (2012) Pupillometry: A window to the preconscious? Perspect Psychol Sci 7: 18–27 10.1177/1745691611427305 26168419

[18] Goldwater BC (1972) Psychological significance of pupillary movements. Psychol Bull 77: 340–355 10.1037/h0032456 5021049

[19] Ukai K (1985) Spatial pattern as a stimulus to the pupillary system. J Opt Soc Am 2: 1094–1100 10.1364/JOSAA.2.001094 4020507

[20] Barbur JL, Harlow AJ, Sahraie A (1992) Pupillary responses to stimulus structure, colour and movement. Ophthalmic Physiol Opt 12: 137–141 10.1111/j.1475-1313.1992.tb00276.x 1408159

[21] Sahraie A, Barbur JL (1997) Pupil response triggered by the onset of coherent motion. Graefes Arch Clin Exp Ophthalmol 235: 494–500 10.1007/BF00947006 9285218

[22] Slooter J, van Norren D (1980) Visual acuity measured with pupil responses to checkerboard stimuli. Invest Ophthalmol Vis Sci 19: 105–108. 7350128

[23] Barbur JL (1995) A study of pupil response components in human vision. In: Ikeda H, Robbins JG, Djamgoz MBA, Taylor A, editors. Basic and Clinical Perspectives in Vision Research: A Celebration of the Career of Hisako Ikeda. New York, NY: Plenum Press.

[24] Laeng B, Endestad T (2012) Bright illusions reduce the eye’s pupil. Proc Natl Acad Sci 109: 2162–2167 10.1073/pnas.1118298109 22308422 PMC3277565

[25] Lowe SW, Ogle KN (1966) Dynamics of the pupil during binocular rivalry. Arch Ophthalmol 75: 395 10.1001/archopht.1966.00970050397017 5903827

[26] Fahle MW, Stemmler T, Spang KM (2011) How much of the “unconscious” is just pre-threshold? Front Hum Neurosci 5. 10.3389/fnhum.2011.00120 PMC3198031 22025912

[27] Naber M, Frässle S, Einhäuser W (2011) Perceptual rivalry: Reflexes reveal the gradual nature of visual awareness. PloS ONE 6: e20910 10.1371/journal.pone.0020910 21677786 PMC3109001

[28] Brenner R, Charles ST, Flynn JT (1969) Pupillary responses in rivalry and amblyopia. Arch Ophthalmol 82: 23–29 10.1001/archopht.1969.00990020025007 5791500

[29] Bárány EH, Halldén U (1948) Phasic inhibition of the light reflex of the pupil during retinal rivalry. J Neurophysiol 11: 25–30. 18921402 10.1152/jn.1948.11.1.25

[30] Harms H (1937) Ort und Wesen der Bildhemmung bei Schielenden. Graefes Arch Clin Exp Ophthalmol 138: 149–210 10.1007/BF01854538 5897590

[31] Naber M, Nakayama K (2013) Pupil responses to high-level image content. J Vis 13: e7 10.1167/13.6.7 23685390

[32] Binda P, Pereverzeva M, Murray SO (2013) Pupil constrictions to photographs of the sun. J Vis 13: e8 10.1167/13.6.8 23685391

[33] Binda P, Pereverzeva M, Murray SO (2013) Attention to bright surfaces enhances the pupillary light reflex. J Neurosci 33: 2199–2204 10.1523/JNEUROSCI.3440-12.2013 23365255 PMC6619119

[34] Posner MI (1980) Orienting of attention. Q J Exp Psychol 32: 3–25 10.1080/00335558008248231 7367577

[35] Baayen RH, Davidson DJ, Bates DM (2008) Mixed-effects modeling with crossed random effects for subjects and items. J Mem Lang 59: 390–412 10.1016/j.jml.2007.12.005

[36] Wyatt HJ (1995) The form of the human pupil. Vision Res 35: 2021–2036 10.1016/0042-6989(94)00268-Q 7660606

[37] Treisman AM, Gelade G (1980) A feature-integration theory of attention. Cognit Psychol 12: 97–136 10.1016/0010-0285(80)90005-5 7351125

[38] Wolfe JM (1994) Guided search 2.0 A revised model of visual search. Psychon Bull Rev 1: 202–238 10.3758/BF03200774 24203471

[39] Buschman TJ, Miller EK (2009) Serial, covert shifts of attention during visual search are reflected by the frontal eye fields and correlated with population oscillations. Neuron 63: 386–396 10.1016/j.neuron.2009.06.020 19679077 PMC2758537

[40] Wierda SM, van Rijn H, Taatgen NA, Martens S (2012) Pupil dilation deconvolution reveals the dynamics of attention at high temporal resolution. Proc Natl Acad Sci 109: 8456–8460 10.1073/pnas.1201858109 22586101 PMC3365158

[41] Zylberberg A, Oliva M, Sigman M (2012) Pupil dilation: A fingerprint of temporal selection during the “Attentional Blink.” Front Psychol 3. 10.3389/fpsyg.2012.00316 PMC3428810 22973248

[42] Mathôt S, Theeuwes J (2011) Visual attention and stability. Philos Trans R Soc B Biol Sci 366: 516–527 10.1098/rstb.2010.0187 PMC3030830 21242140

[43] Carrasco M (2011) Visual attention. Vision Res 51: 1484–1525 10.1016/j.visres.2011.04.012 21549742 PMC3390154

[44] Carrasco M, Ling S, Read S (2004) Attention alters appearance. Nat Neurosci 7: 308–313 10.1038/nn1194 14966522 PMC3882082

[45] Mack A, Rock I (1998) Inattentional Blindness: Perception Without Attention. Cambridge, MA: MIT Press.

[46] Reynolds JH, Pasternak T, Desimone R (2000) Attention increases sensitivity of V4 neurons. Neuron 26: 703–714 10.1016/S0896-6273(00)81206-4 10896165

[47] Spitzer H, Desimone R, Moran J (1988) Increased attention enhances both behavioral and neuronal performance. Science 240: 338–340 10.1126/science.3353728 3353728

[48] Broadbent DE (1958) Perception and Communication. London: Pergamon Press.

[49] Porter G, Troscianko T, Gilchrist ID (2007) Effort during visual search and counting: Insights from pupillometry. Q J Exp Psychol 60: 211–229 10.1080/17470210600673818 17455055

[50] Jainta S, Vernet M, Yang Q, Kapoula Z (2011) The pupil reflects motor preparation for saccades - even before the eye starts to move. Front Hum Neurosci 5. 10.3389/fnhum.2011.00097 PMC3202225 22046154

[51] Rayner K (1998) Eye movements in reading and information processing: 20 years of research. Psychol Bull 124: 372–422 10.1037/0033-2909.124.3.372 9849112

[52] Kowler E (2011) Eye movements: The past 25 years. Vision Res 51: 1457–1483 doi:16/j.visres.2010.12.014 21237189 10.1016/j.visres.2010.12.014 PMC3094591

[53] Mathôt S (2013) Visual Attention and Stability [PhD thesis]. Amsterdam: VU University. Available: 10.6084/m9.figshare.704402.

[54] Walls GL (1962) The evolutionary history of eye movements. Vision Res 2: 69–80 10.1016/0042-6989(62)90064-0

[55] Land MF (1999) Motion and vision: Why animals move their eyes. J Comp Physiol A Neuroethol Sens Neural Behav Physiol 185: 341–352 10.1007/s003590050393 10555268

[56] Netser S, Ohayon S, Gutfreund Y (2010) Multiple manifestations of microstimulation in the optic tectum: eye movements, pupil dilations, and sensory priming. J Neurophysiol 104: 108–118 10.1152/jn.01142.2009 20427617

[57] Krauzlis RJ (2004) Recasting the smooth pursuit eye movement system. J Neurophysiol 91: 591–603 10.1152/jn.00801.2003 14762145

[58] Robinson DA (1968) Eye movement control in primates. Science 161: 1219–1224 10.1126/science.161.3847.1219 5302604

[59] Kastner S, De Weerd P, Desimone R, Ungerleider LG (1998) Mechanisms of directed attention in the human extrastriate cortex as revealed by functional MRI. Science 282: 108–111 10.1126/science.282.5386.108 9756472

[60] Mathôt S, Schreij D, Theeuwes J (2012) OpenSesame: An open-source, graphical experiment builder for the social sciences. Behav Res Methods 44: 314–324 10.3758/s13428-011-0168-7 22083660 PMC3356517

[61] Peirce JW (2007) PsychoPy: Psychophysics software in Python. J Neurosci Methods 162: 8–13 10.1016/j.jneumeth.2006.11.017 17254636 PMC2018741

[62] Mathôt S (2013) A Simple Way to Reconstruct Pupil Size During Eye Blinks. Available: 10.6084/m9.figshare.688002.

[63] Engbert R, Kliegl R (2003) Microsaccades uncover the orientation of covert attention. Vision Res 43: 1035–1045 10.1016/S0042-6989(03)00084-1 12676246

